# Secretome Profiling of *Lactiplantibacillus plantarum* CRL681 Predicts Potential Molecular Mechanisms Involved in the Antimicrobial Activity Against *Escherichia coli* O157:H7

**DOI:** 10.3390/antibiotics15010096

**Published:** 2026-01-17

**Authors:** Ayelen Antonella Baillo, Leonardo Albarracín, Eliana Heredia Ojeda, Mariano Elean, Weichen Gong, Haruki Kitazawa, Julio Villena, Silvina Fadda

**Affiliations:** 1 Reference Center for Lactobacilli (CERELA-CONICET), San Miguel de Tucuman CP4000, Tucuman, Argentina; 2 Laboratory of Respiratory Immunology (LaRI), Division of Animal Immunology and Omics, International Education and Research Center for Food and Agricultural Immunology (CFAI), Graduate School of Agricultural Science, Tohoku University, Sendai 980-8572, Japan; 3 Food and Feed Immunology Group, Laboratory of Animal Food Function, Graduate School of Agricultural Science, Tohoku University, Sendai 980-8572, Japan; 4 Livestock Immunology Unit, International Education and Research Centre for Food and Agricultural Immunology (CFAI), Graduate School of Agricultural Science, Tohoku University, Sendai 980-8572, Japan

**Keywords:** *Lactiplantibacillus plantarum*, secretome, exoproteome, surfaceome, antimicrobial activity, *Escherichia coli* O157:H7

## Abstract

**Background/Objectives.** *Lactiplantibacillus plantarum* CRL681 has previously demonstrated a strong antagonistic effect against *Escherichia coli* O157:H7 in food matrices; however, the molecular mechanisms underlying this activity remain poorly understood. Since initial interactions between beneficial bacteria and pathogens occur mainly at the cell surface and in the extracellular environment, the characterization of the bacterial secretome is essential for elucidating these mechanisms. In this study, the secretome of *L. plantarum* CRL681 was comprehensively characterized using an integrated in silico and in vitro approach. **Methods.** The exoproteome and surfaceome were analyzed by LC-MS/MS under pure culture conditions and during co-culture with *E. coli* O157:H7. Identified proteins were functionally annotated, classified according to subcellular localization and secretion pathways, and evaluated through protein–protein interaction network analysis. **Results.** A total of 275 proteins were proposed as components of the CRL681 secretome, including proteins involved in cell surface remodeling, metabolism and nutrient transport, stress response, adhesion, and genetic information processing. Co-culture with EHEC induced significant changes in the expression of proteins associated with energy metabolism, transport systems, and redox homeostasis, indicating a metabolic and physiological adaptation of *L. plantarum* CRL681 under competitive conditions. Notably, several peptidoglycan hydrolases, ribosomal proteins with reported antimicrobial activity, and moonlighting proteins related to adhesion were identified. **Conclusions.** Overall, these findings suggest that the antagonistic activity of *L. plantarum* CRL681 against *E. coli* O157:H7 would be mediated by synergistic mechanisms involving metabolic adaptation, stress resistance, surface adhesion, and the production of non-bacteriocin antimicrobial proteins, supporting its potential application as a bioprotective and functional probiotic strain.

## 1. Introduction

Lactic acid bacteria (LAB), particularly the species *Lactiplantibacillus plantarum*, have generated considerable interest in recent years due to their relevance as probiotics and as biopreservation agents in food [1,2,3]. These bacteria can promote a healthy intestinal microbiota balance and inhibit the development of foodborne pathogens through various mechanisms, including the production of antimicrobial metabolites, competition for nutrients and adhesion sites, and modulation of the immune system [4]. This antagonistic capacity of LAB is particularly important against foodborne pathogenic microorganisms, which represent a significant public health risk. Among these, enterohemorrhagic *Escherichia coli* (EHEC) is one of the primary causative agents of hemorrhagic colitis and hemolytic uremic syndrome (HUS), which can even result in death [5]. Within this group, *E. coli* O157:H7 is characterized by an extremely low infectious dose and a high capacity to induce damage in the host [6]. Currently, the treatment of Shiga toxin-producing *E. coli* (STEC)-HUS is mainly supportive, including fluid resuscitation, correction of electrolyte imbalances, and control of hypertension [7]. The use of antibiotics in this condition is controversial and, at present, is not recommended. Therefore, preventive measures, particularly in food safety control, are of fundamental importance in preventing the spread of infection [7]. In this context, the extensive use of antimicrobials in animal husbandry has played an important role in the emergence and dissemination of multidrug-resistant strains exhibiting resistance to critical antimicrobials, such as extended-spectrum β-lactamases, carbapenems, colistin, and tigecycline [8,9].

Previous studies of our group demonstrated that certain LAB strains are capable of inhibiting the growth of pathogenic *E. coli* in both meat matrices and processing surfaces, as well as in murine infection models [10,11,12]. Among the strains studied, *Lactiplantibacillus plantarum* CRL681 showed a notable inhibitory effect against EHEC. The nature of this inhibition was evaluated through agar diffusion assays and inhibition curves in a meat model system [13,14], concluding that the antimicrobial effect manifests only when the strain is viable and metabolically active. Moreover, it was determined that the inhibition is not primarily due to lactic acid or bacteriocin production, as these antimicrobial peptides are generally ineffective against Gram-negative bacteria [15] and *L. plantarum* CRL681 lacks genes associated with their synthesis [16]. The inhibitory effect of the CRL681 strain was also analyzed in different meat matrices (ground meat and meat discs), showing a bacteriostatic effect on EHEC, along with reduced adherence and colonization of surfaces [10]. In addition, *L. plantarum* CRL681 was shown to possess immunomodulatory functions and a protective effect against the infection caused by enterotoxigenic *E. coli* (ETEC) in mice [11].

Considering that initial interactions between beneficial bacteria and pathogens primarily occur at the cellular surface and intercellular space, the study of extracellular and membrane-associated proteins in strains such as *L. plantarum* CRL681 is of fundamental importance to understand the molecular mechanisms underlying their antagonistic effects. Surface proteins and secreted proteins constitute the bacterial secretome, which comprises the exoproteome and surfaceome [17,18,19]. This protein repertoire is essential for bacterial survival and competitiveness, participating in processes such as nutrient acquisition, surface adhesion, antimicrobial compound production, biofilm formation, and immune modulation [20,21,22,23,24]. In this context, extracellular and surface-associated proteins of LAB have been described as playing a key role in adaptation and interaction with the environment [25,26], and determining their functional properties [27]. Therefore, understanding the composition and functional roles of the *L. plantarum* CRL681 secretome is essential for clarifying the mechanisms underlying its inhibitory activity against EHEC and for enhancing its use in food biotechnology.

Based on this background, the present study focused on investigating the mechanisms that confer *L. plantarum* CRL681 the ability to inhibit EHEC. For this purpose, genomic and proteomic characterizations of the CRL681 strain were performed, with a particular emphasis on the secretome. The studies identify potential proteins involved in its pathogen-inhibitory capacity and its interaction with host cells.

## 2. Results

### 2.1. In Silico Analysis of L. plantarum CRL681 Secretome

The in silico analysis of *L. plantarum* CRL681 secretome was performed using its previously sequenced genome [16]. The genome of the reference bacterium of this species, *L. plantarum* WCFS1 [28], was used for comparisons. It has been reported that the WCFS1 strain encodes a total of 313 extracellular proteins [28]; therefore, our analysis focused on the identification and comparison of extracellular proteins predicted in silico in both strains. BLASTp was used to determine the presence or absence of the coding sequences of the extracellular proteins described in the WCFS1 strain within the genome of *L. plantarum* CRL681, evaluating the percentage of identity and coverage. As a result, 274 proteins were identified in the genome of *L. plantarum* CRL681 that would be part of the in silico secretome (Table S1). These proteins represent approximately 8.7% of the total coding genome of this strain, a value slightly lower than the 10% reported for the in silico secretome of *L. plantarum* WCFS1, composed of 313 proteins [28].

**Figure 1:**
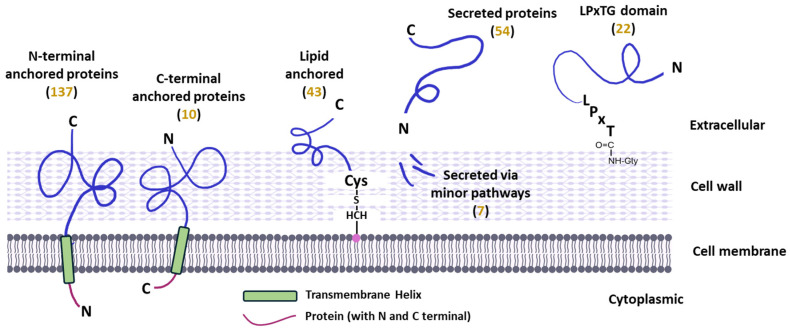
Representation of the extracellular proteins identified in silico in *Lactiplantibacillus plantarum* CRL681 according to their type of anchoring and secretion. The number of proteins in each category are shown between brackets.

The comparison between the two strains suggests that, although they have a similar genome size (3,370,224 bp in *L. plantarum* CRL681 and 3,348,624 bp in the WCFS1 strain), the differences in the number of protein-coding sequences (3081 in *L. plantarum* CRL681 and 3015 in the WCFS1 strain) could influence the composition and functionality of their respective secretomes [16,28]. The predicted proteins were classified according to their anchoring and secretion mechanism (Figure 1).

A total of 137 proteins anchored to the membrane via an N-terminal end, 10 proteins with C-terminal anchoring, and 43 proteins linked via a lipid bond were identified. In addition, 54 extracellular proteins and 7 proteins secreted by minor pathways were registered (Figure 1, Table S1). In addition, 22 proteins anchored to the cell wall via the LPxTG motif were identified. As for the proteins present only in the WCFS1 strain and absent in *L. plantarum* CRL681, several correspond to prophage proteins, proteins involved in the synthesis and transport of plantaricin, and proteins related to adhesion, including those with LPXTG motifs, specific adhesins, and proteins from the CscA/B/C and ErfK families.

### 2.2. In Vitro Study of L. plantarum CRL681 Exoproteome

**Figure 2:**
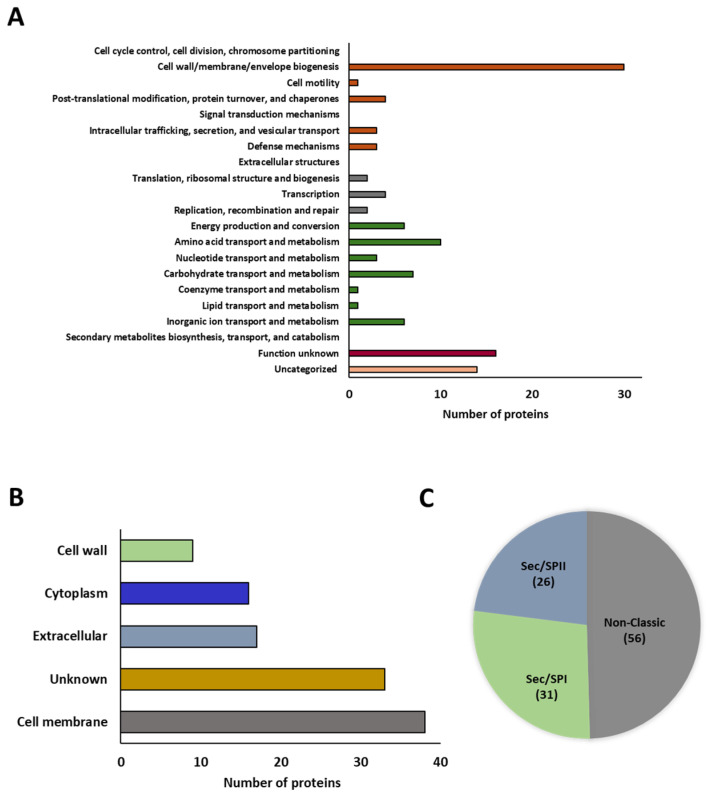
In vitro characterization of *Lactiplantibacillus plantarum* CRL681 exoproteome. (**A**) Functional distribution of the exoproteome proteins of *L. plantarum* CRL681 according to the Clusters of Orthologous Genes (COG) database. The different colors represent groups of specific functional categories: proteins related to cellular processes and signaling (orange), associated with information processing and storage (grey), involved in cellular metabolism (green) and proteins of unknown (burgundy) or uncategorized (pink) functions. (**B**) PSORT-predicted cellular localization of the exoproteome of *L. plantarum* CRL681. (**C**) Analysis of the secretion pathways involved in the proteins identified in *L. plantarum* CRL681 using the SignalP program.

During the culture of *L. plantarum* CRL681 in a chemically defined medium (CDM) at 30 °C for 24 h, 113 proteins were identified in the exoproteome (Table S2), which were functionally classified according to their COG category. As shown in Figure 2A, 41 proteins were grouped into the category of “cell and signaling processes”, of which 30 are directly related to cell wall and/or membrane biogenesis. It should be noted that many of these enzymes are peptidoglycan hydrolases. The second-largest group of proteins corresponds to “metabolic processes” with 34 proteins involved in various metabolite transport processes. A total of 8 proteins were classified in the “information storage and processing” category and 30 proteins could not be characterized or have unknown functions.

The subcellular locations of the 113 proteins identified in the exoproteome of *L. plantarum* CRL681 were analyzed using the PSORT server. The proteins were classified into five groups (Figure 2B): 38 proteins were located in the cell membrane, 16 in the cytoplasm, 9 in the cell wall, and 17 were extracellular. On the other hand, 33 proteins could not be classified and were grouped under the category of unknown location. Additionally, the SignalP program was used to determine the secretion pathways used by the identified proteins (Figure 2C). These proteins were classified according to the type of peptidase that cleaves the signal peptide: signal peptidase I (SPase I, Sec/SPI) or signal peptidase II (SPase II, Sec/SPII), the latter specialized in the secretion of lipoproteins for their incorporation into the cell membrane. Thirty-one and 26 proteins were detected for Sec/SPI and Sec/SPII, respectively. In addition, 56 proteins were identified as secreted by non-classical pathways (Figure 2C).

It should be noted that among the 16 proteins identified as cytoplasmic, 9 are considered moonlighting proteins. These proteins lack a signal peptide for secretion and are therefore secreted into the medium by non-classical pathways [29]. Table 1 lists the moonlighting proteins identified in the exoproteome of *L. plantarum* CRL681. It was observed that most of them have a secondary function related to adhesion, according to previously published data in other LAB strains.

### 2.3. Analysis of L. plantarum CRL681 Exoproteome in the Presence of EHEC

In the next set of experiments, *L. plantarum* CRL681 was co-cultured with *E. coli* O157:H7 NCTC12900 and the exoproteome was analyzed. It was found that 25 proteins from the exoproteome of the lactic acid bacterium showed reduced expression after 24 h compared to the control culture (*L. plantarum* CRL681 in the absence of EHEC) (Table S3). Interestingly, no overexpressed proteins were identified under these conditions. The proteins that showed decreased expression presented changes between 1.9 and 8.2 folds. Among the negatively regulated proteins, 6 were identified as ABC-type transporters, 6 had enzymatic activity, and 8 had unknown functions. Among the enzymes, 3 are involved in protein hydrolysis, 1 in thiamine synthesis, and 1 in peptidoglycan cross-linking. In addition, 3 cytoplasmic proteins associated with energy metabolism and exhibiting moonlighting function were identified: 1 with transcriptional regulatory function and 1 other involved in cell adhesion.

### 2.4. Interaction Network of L. plantarum CRL681 Exoproteome

**Figure 3:**
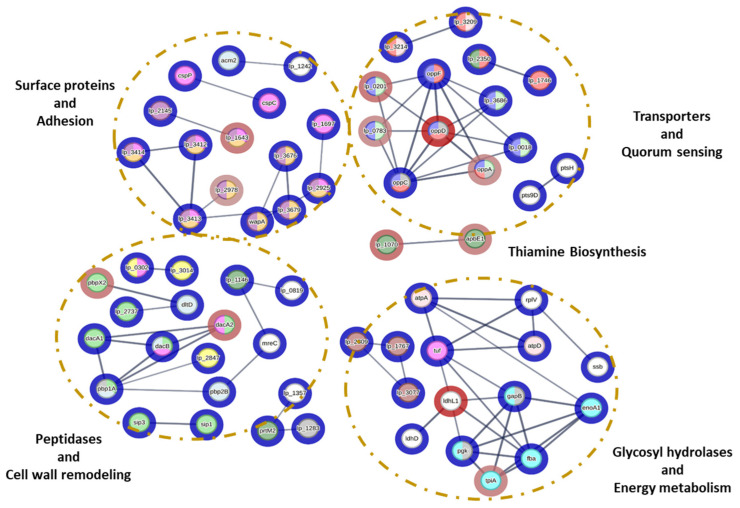
Interaction network of proteins identified in the exoproteome of *Lactiplantibacillus plantarum* CRL681 during the co-culture with *Escherichia coli* O157:H7 NCTC12900. The colors in the node halos indicate a decrease in protein expression (red) or no changes (blue). The intensity of the color indicates the level of expression. In addition, the different colors in the nodes represent distinct functionality: blue nodes (Proteins related to metabolite transport); red nodes (Quorum sensing and Stress response); fuchsia nodes (Adhesion proteins); violet and orange nodes (surface proteins); green nodes (Peptidases); yellow nodes (Proteins with LysM domain); brown nodes (glycosylhydrolases); light blue nodes (proteins involved in energy generation); dark green nodes (Thiamine synthesis); light gray nodes (Proteins with probiotic function).

The interactions of *L. plantarum* CRL681 proteins expressed in the presence/absence of *E. coli* O157:H7 were evaluated (Figure 3). A total of 65 proteins presented different types of interactions. Interactions were identified between key proteins involved in various biological functions. Those related to metabolite transport are essential for the uptake of nutrients necessary for the optimal growth of *L. plantarum* CRL681. Some of these proteins are also involved in quorum sensing signal detection and environmental stress response. Notably, four proteins (oppD, oppA, lp_0783, lp_0201) belonging to these functional clusters exhibited significantly decreased abundance during co-culture with *E. coli.* In addition, STRING analysis revealed associations between three glycosyl hydrolases (lp_2809, lp_1767, lp_3077) involved in carbohydrate degradation and a relevant set of proteins linked to sugar catabolism and energy production. This group included glyceraldehyde 3-phosphate dehydrogenase (GapB), phosphoglycerate kinase (Pgk), and lactate dehydrogenase (LdhL), although only LdhL showed a statistically significant reduction in abundance in the co-culture condition. These proteins were related to the translation elongation factor Tu (tuf), which also interacts with ATPases (atpA and atpD) and is linked to cell adhesion. On the other hand, proteins linked to thiamine biosynthesis like apbE1 and Lp_1070 that decreased in the co-culture showed only interactions between themselves (Figure 3). This could suggest an adaptation to a competitive environment through regulation of resource use, probably aimed at optimizing nutrient utilization under stress conditions and thus regulating metabolic efficiency.

Some interactions were also observed in proteins associated with cell wall remodeling. Most of these proteins did not show obvious expression changes in the presence of EHEC. However, a lower expression of peptidases such as serine D-Ala-D-Ala carboxypeptidase (pbpX2, dacA) was noted during the co-culture. An interaction was also found between the peptidases sip3 and sip1, which have the function of removing hydrophobic N-terminal signal peptides when proteins are translocated across membranes. In addition, interactions between surface proteins with different cell wall anchoring domains were observed. The stress response proteins CspP and CspC, related to each other and unaffected in the presence of EHEC, have been described as moonlighting proteins that function as adhesins to epithelial cells [38]. This group of proteins stands out for its possible role in the interaction of *L. plantarum* CRL681 with the environment and microbial competition, possibly regulating metabolism to mitigate environmental stress and promoting survival in adverse conditions.

### 2.5. In Vitro Analysis of L. plantarum CRL681 Surfaceome

**Figure 4:**
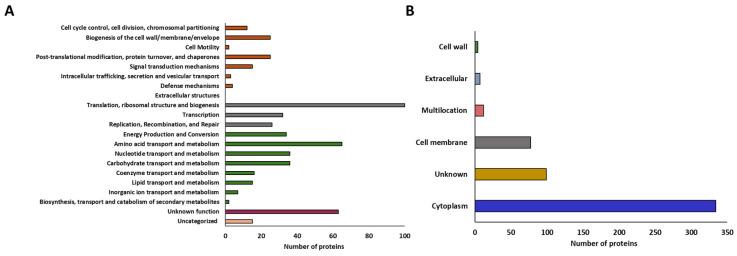
In vitro characterization of *Lactiplantibacillus plantarum* CRL681 surfaceome. (**A**) Functional distribution of the surfaceome proteins of *L. plantarum* CRL681 according to the Clusters of Orthologous Genes (COG) database. The different colors represent groups of specific functional categories: proteins related to cellular processes and signaling (orange), associated with information processing and storage (grey), involved in cellular metabolism (green) and proteins of unknown (burgundy) or uncategorized (pink) functions. (**B**) PSORT-predicted cellular localization of the surfaceome of *L. plantarum* CRL681.

The analysis of the surfaceome identified 533 proteins (Table S4), which were functionally classified according to their COG category. As shown in Figure 4A, 211 proteins were grouped into the categories of “metabolic processes,” including “metabolite transport,” “energy production,” and “biosynthesis, transport, and catabolism of secondary metabolites.” In addition, 158 proteins were assigned to the “information processing and storage” group, within which 100 proteins were classified in the “translation, ribosomal structure, and biogenesis” category. On the other hand, 86 proteins were included in the category related to “signaling and cellular processes”, 63 had an unknown function, and 15 could not be categorized.

The subcellular locations of the 533 proteins identified in the surfaceome of *L. plantarum* CRL681 were analyzed in silico using the PSORT server. The proteins were classified into five groups: 334 were located in the cytoplasm, 77 in the cell membrane, 4 in the cell wall, and 12 proteins were classified as multi-localized (Figure 4B). In addition, 7 proteins were identified as extracellular and 99 were classified as unknown localization. Among the cytoplasmic proteins identified in the analysis, 23 are known to be exported to the cell surface, where they perform secondary functions. The identified moonlighting proteins are described in Table 2.

### 2.6. Analysis of L. plantarum CRL681 Surfaceome in Co-Culture with EHEC

We next aimed to evaluate the changes in the surfaceome of the CRL681 strain in co-culture with EHEC. The presence of the pathogen in the culture medium induced a decrease in the expression of 15 proteins and the overexpression of 2 in *L. plantarum* CRL681 (Table S5). The changes in expression levels of the downregulated proteins ranged from 1.5- to 8-fold. Among the 15 proteins with reduced expression, one ABC-type transporter, one protein with unknown function, and 13 proteins with enzymatic activity were identified. Of these enzymes, two are involved in carbohydrate hydrolysis, three in stress response, one in nucleotide biosynthesis, one in amino acid synthesis, one in energy production, one in phosphate hydrolysis, and one in RNA processing, while one protein was identified as a protease and two as ribosomal proteins. In addition, two overexpressed proteins with enzymatic functions were detected: glutathione reductase and acetate kinase (Table S5).

### 2.7. Interaction Network of L. plantarum CRL681 Surfaceome Proteins

**Figure 5:**
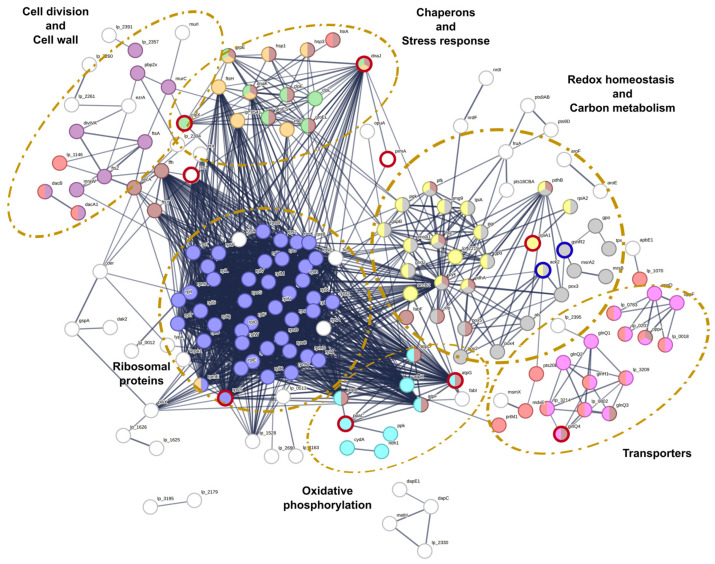
Interaction network of proteins identified in the surfaceome of *Lactiplantibacillus plantarum* CRL681 during the co-culture with *Escherichia coli* O157:H7 NCTC12900. The colors in the node halos indicate a decrease (red) or increase (blue) in protein expression. The intensity of the color indicates the level of expression. In addition, the different colors in the nodes represent distinct functionality: blue nodes (ribosomal proteins), gray nodes (redox homeostasis), yellow nodes (carbon metabolism), magenta and lilac nodes (ABC-type transporters), light blue nodes (oxidative phosphorylation), light green and orange nodes (chaperones and stress response proteins), purple nodes (cell division and cell wall formation), and brown nodes (proteins identified as stress response through bibliographic sources).

Analysis of the interaction networks between the surfaceome proteins identified in *L. plantarum* CRL681 in co-culture with *E. coli* O157:H7 showed that ribosomal proteins constitute the central core of the interactome network, which is expected because of their fundamental role in the translation machinery (Figure 5, high-resolution figure is provided as Supplementary Figure S1). On the other hand, proteins involved in redox homeostasis, such as glutathione reductase (GshR2), showed overexpression in co-culture with EHEC. GshR2 interacts with enzymes such as methionine S-oxide reductase (MsrA2 and MsrB) and thiol peroxidase (Gpo, Tpx), suggesting an adaptive response to oxidative stress generated by bacterial interaction. These proteins play an essential role in the repair of proteins damaged by oxidation and in the detoxification of peroxides. In carbon metabolism, the overexpression of acetate kinase (Ack2), which interacts with several pyruvate oxidases (Pox2, Pox3, Pox4, and Pox5), was notable. These enzymes are key to aerobic growth and the generation of acetyl phosphate as an energy intermediate. A decrease in the expression of ribose-5-phosphate epimerase (RpiA), involved in the generation of metabolic precursors, was also observed.

A decrease in the expression of the glutamine/histidine ABC transporter (GlnQ4) was observed, and no significant alterations were detected in the expression of other transporters in this family, suggesting a specific regulation of specific amino acid assimilation pathways, possibly associated with nutrient availability or a competitive response to EHEC. On the other hand, decreases in ATP synthase gamma chain (AtpG) and inorganic pyrophosphatase (PpaC) were detected (Figure 5, Supplementary Figure S1), which participate in central pathways of cellular energy generation and utilization: AtpG is essential for ATP synthesis through oxidative phosphorylation, while PpaC catalyzes pyrophosphate (PPi) hydrolysis, contributing both to energy balance control and to the promotion of energy-requiring biosynthetic reactions. The decrease in the abundance of these proteins suggests that *L. plantarum* CRL681 adjusts its energy metabolism in response to environmental conditions generated during interaction with EHEC, possibly characterized by lower oxygen availability or greater competition for resources. A decrease in the abundance of ClpX and DnaJ chaperones was also observed in *L. plantarum* CRL681. These proteins are part of a broader stress response network, interacting with other chaperones such as GroEL, DnaK, and GrpE, which are essential for protection against adverse conditions such as thermal and hyperosmotic stress.

### 2.8. Proposed Secretome for L. plantarum CRL681

**Figure 6:**
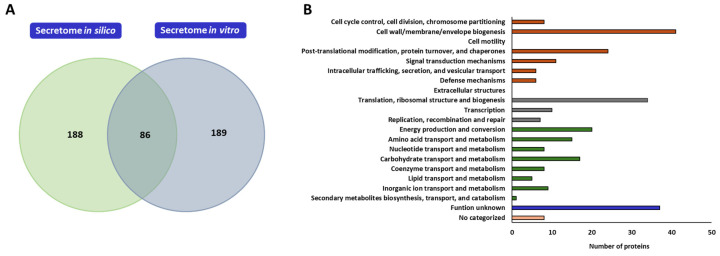
Proposed secretome for *Lactiplantibacillus plantarum* CRL681. (**A**) Venn diagram showing the number of proteins comprising the in silico secretome and the secretome evaluated in vitro for *L. plantarum* CRL681. (**B**) Functional distribution of the proteins in the proposed secretome of *L. plantarum* CRL681 according to the orthologous groups database (COG). The different colors represent groups of specific functional categories: proteins related to cellular processes and signaling (orange), associated with information processing and storage (grey), involved in cellular metabolism (green) and proteins of unknown (burgundy) or uncategorized (pink) functions.

Considering the information obtained through the in silico analysis and the results of the in vitro studies of the exoproteome and surfaceome of *L. plantarum* CRL681, a possible secretome conformation was proposed for this strain. To this end, the identified proteins were manually filtered considering the criteria specified above. A total of 275 proteins were identified that could potentially form part of the secretome of *L. plantarum* CRL681, considering both the exoproteome and the surfaceome (Table S6). A comparison was made between the proteins of *L. plantarum* CRL681 secretome identified in silico and those detected experimentally through in vitro proteomic assays. The Venn diagram shows that only 86 proteins were common in both approaches, while 188 were exclusive to the in silico analysis and 189 were detected only in vitro (Figure 6A). Then, the proposed surfaceome of *L. plantarum* CRL681 contains proteins mainly involved in cellular and signaling processes (96), among which enzymes involved in cell surface remodeling and turnover, chaperones, and proteins involved in stress response stand out (Figure 6B). In addition, 83 proteins involved in metabolism and transport of metabolites essential for growth and 51 proteins related to genetic information processing are proposed. Within the latter group, there are 34 ribosomal proteins, some of which have been described as having an important role in pathogen inhibition [48]. Of note, 37 proteins were classified as having unknown functions and 8 could not be categorized.

### 2.9. Proteins of the Proposed Secretome of L. plantarum CRL681 with Technological and Functional Properties

**Figure 7:**
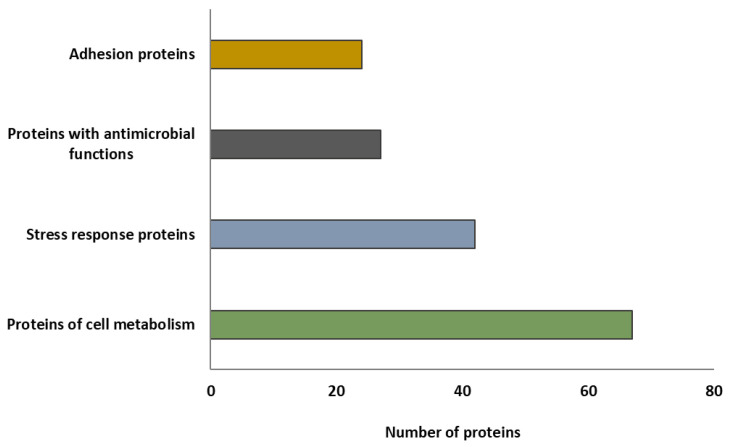
Functional classification of the proteins identified in the secretome of *Lactiplantibacillus plantarum* CRL681. Proteins potentially related to competitive advantages, antagonistic effects, and the ability to establish/adhere to epithelial cells and matrices such as meat were analyzed.

In order to associate the analyses performed in this study with the antimicrobial and probiotic properties of *L. plantarum* CRL681 described in previous works [10,11,14], a group of proteins from the secretome that could be related to competitive advantages, antagonistic effects, and the ability to establish/adhere to epithelial cells and matrices such as meat was selected (Table S7). As shown in Figure 7, the proteins were classified into four specific groups.

The secretome of *L. plantarum* CRL681 would contain 67 proteins involved in cellular metabolic processes, and 42 proteins involved in stress response. In addition, 22 proteins with possible antimicrobial functions were proposed to be part of the secretome according to previous studies [45,46,49,50,51] and 24 proteins related to cell adhesion. The latter group included moonlighting proteins involved in adhesion, when secreted by the cell.

## 3. Discussion

In order to describe the proteins in *L. plantarum* CRL681 that could be involved in its ability to inhibit *E. coli* O157:H7, the secretome of the lactic acid bacterium was studied, taking into account that the initial interactions between beneficial bacteria and pathogens occur at the cell surface and/or in the intercellular space. The in vitro analysis of the secretome was performed by dividing the proteome into two fractions: the exoproteome and the surfaceome. These data were complemented by an in silico analysis of the CRL681 strain genome.

The comparative analysis between the in silico (genomic) and in vitro (proteomic) secretome of *L. plantarum* CRL681 revealed differences that show the advantages and limitations of each approach. The in silico analysis predicted 274 proteins and the in vitro proteomic study predicted 275, of which only 86 were common, while more than 180 were unique to each methodology. The in silico approach allows the prediction of potentially secreted proteins but does not reflect gene expression or regulation under specific conditions, which requires complementation with proteomics to develop a more comprehensive overview. In turn, in vitro analysis shows the proteins that are actually present in a determinate condition, including the moonlighting ones that cannot be detected by bioinformatic predictions, as they do not follow classical secretion pathways. Thus, proteomics reveals proteins that are dependent on factors such as stress or culture medium, while the in silico analysis remains useful when the proteome is difficult to obtain or highly variable. Therefore, both approaches are necessary for a complete characterization of a secretome.

The results of this study allow us to propose a secretome for *L. plantarum* CRL681 by identifying proteins with putative relevant functions for the competitive advantage against EHEC demonstrated in previous studies [10,14]. The proposed secretome for *L. plantarum* CRL681 includes 275 proteins with functions related to the strain’s environmental adaptation and antimicrobial potential. From a functional perspective, they can be grouped into three main categories: (a) cell surface remodeling and stress response, (b) metabolism and transport, and (c) genetic information processing. In the first group (a), 96 proteins involved in cellular and signaling processes were identified, suggesting active regulation of cell architecture and homeostasis. Of these, 41 participate in cell wall and membrane biogenesis, some of which may act as antagonistic factors against pathogens. On the other hand, in group (b), 83 proteins related to the metabolism and transport of essential metabolites were identified, reinforcing the assumption that *L. plantarum* CRL681 optimizes its energy and carbon resources according to its environment. The presence of transporters, mostly of the ABC type and phosphotransferase systems (PTS), highlights the importance of nutrient uptake mechanisms in the regulation of bacterial metabolism. In addition, the enzymes with hydrolytic activity identified in the secretome, including carbohydrate hydrolases, could participate in the degradation of complex polymers, facilitating access to carbon sources. As for group (c), 51 proteins were identified, including 34 ribosomal proteins. On the other hand, analysis of secretion pathways showed that 122 proteins lack a characterized export mechanism, suggesting the existence of yet undescribed routes or passive release following cell lysis. However, 100 proteins secreted by non-classical mechanisms were identified, including moonlighting proteins, in addition to those exported by Sec/SPI (31 proteins) and Sec/SPII (26 proteins) signal peptide-dependent pathways.

**Figure 8:**
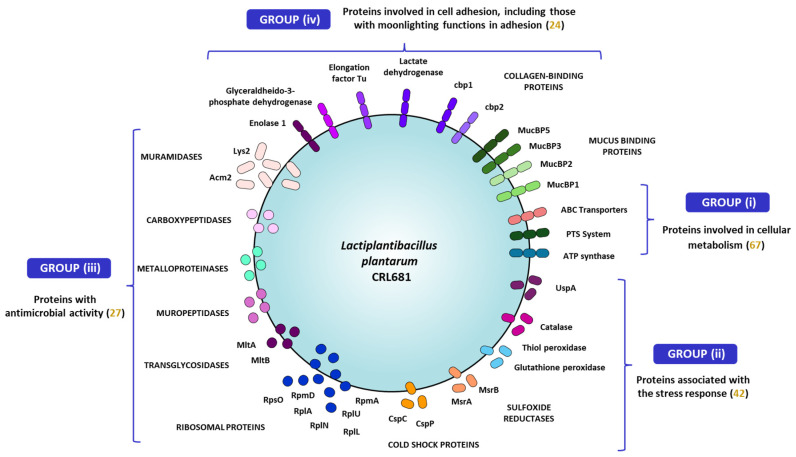
Graphical representation of the proposed *Lactiplantibacillus plantarum* CRL681 secretome, showing the proteins involved in the competitive advantage against EHEC. The numbers in brackets indicate the number of proteins in each category.

Among the proteins that constitute the secretome of *L. plantarum* CRL681, those that could give it a competitive advantage and might explain its antagonistic capacity against EHEC can be grouped in four categories as depicted in Figure 8.

The group (i) is constituted by proteins involved in cellular metabolism (67 proteins), including transporters and enzymes essential for energy generation. These proteins would allow *L. plantarum* CRL681 to optimize nutrient uptake and utilization, which is key to its growth and survival in competitive environments. High efficiency in resource acquisition highlights the ability of *L. plantarum* to thrive in challenging ecological niches. Proteins in the group (ii) are associated with stress response (42 proteins), which would allow *L. plantarum* CRL681 to adapt and respond effectively to adverse environmental conditions, maintaining its competitiveness against other microorganisms. This group includes proteins that respond to low temperatures or the presence of reactive oxygen species, among others. The expression of stress-related and quorum sensing proteins suggests that the CRL681 strain possesses detection and adaptation mechanisms that allow it to modulate its metabolism and behavior in response to environmental changes. Group (iii) includes proteins with antimicrobial activity, such as peptidoglycan hydrolases and ribosomal proteins. For this group, only proteins whose antimicrobial activity has been reported in the literature were included, in addition to the 50S L22 ribosomal protein. Although there are no bibliographic references to support its antimicrobial function, this protein was detected in both the exoproteome and the in vitro surfaceome of the CRL681 strain, suggesting a possible implication in interaction with the pathogen. Considering that these proteins were found in high abundance in *L. plantarum* CRL681, they are likely to contribute to the antagonistic effect observed against EHEC, as discussed below. Finally, group (iv) comprises proteins involved in cell adhesion, which include those with moonlighting functions in adhesion. These proteins would play a crucial role in the ability of *L. plantarum* CRL681 to colonize different ecological niches.

In accordance with data reported for the secretomes of other lactobacilli [52,53,54], the presence of multiple proteins with cell wall hydrolytic functions was noted in *L. plantarum* CRL681. Peptidoglycan, in addition to its structural role, is a target of peptidoglycan hydrolases, enzymes that participate in cell wall remodeling and can exert antimicrobial, adhesive, and immunomodulatory functions [55,56,57]. In this study, LysM domain transglycosidases, NlpC/P60 domain muropeptidases, D-Ala-D-Ala serine-type carboxypeptidases, Zn^2+^-dependent metalloproteases, and lysozymes were identified, all of which are potentially involved in the degradation of the bacterial cell wall. Similar results were reported for *L. acidophilus* [36], *L. johnsonii* [50], *L. reuteri*, *L. rhamnosus* [51], *Ligilactobacillus murinus* [58] as well as other strains of the *L. plantarum* species [59]. In these works, the enzymes were associated with the inhibition of Gram-positive and Gram-negative pathogens, such as *Salmonella*, *E. coli*, and *Klebsiella pneumoniae*.

Protocols based on protein identification using liquid chromatography coupled with mass spectrometry (LC-MS/MS) allow for the identification of a higher proportion of low-abundance and low-solubility proteins, such as those associated with the surfaceome, compared to gel-based approaches [60,61]. However, minimal cell lysis (<2%) can introduce cytoplasmic proteins into the surfaceome fractions [23,62]. Nevertheless, several studies argue that their presence on the surface may also be due to non-classical secretion mechanisms, which allow the export of proteins without a signal peptide, known as moonlighting [17,63]. In this study, the viability of *L. plantarum* CRL681 was verified before and after the use of trypsin during the extraction of surfaceome proteins (Table S8), with a slight reduction in cell counts observed. However, the cell concentration remained within the same order of magnitude after cell digestion, so the vast majority of the proteins detected would correspond mainly to the surfaceome of *L. plantarum* CRL681, although the presence of some cytoplasmic proteins resulting from cell lysis cannot be discarded. In this context, the analysis of *L. plantarum* CRL681 surfaceome revealed approximately 50 ribosomal proteins. Although traditionally associated with protein synthesis, some ribosomal proteins have antimicrobial activity and are considered an emerging type of antimicrobial peptides (AMPs) [64,65]. Cisneros et al. [66] identified a high number of ribosomal proteins that were upregulated during mixed biofilm growth of *Pediococcus pentosaceus* CRL2145 with EHEC, also suggesting their involvement in environmental sensing and antimicrobial activities. These AMPs can destabilize bacterial membranes or induce the generation of reactive oxygen species, affecting essential structures and promoting cell death [67,68]. Our analysis identified 50 ribosomal proteins in *L. plantarum* CRL681; however, according to the literature available [44,45,46,49,69,70], only eight of them have been proven to have antimicrobial activity.

In LAB, the antagonistic activity of ribosomal proteins against pathogens has been demonstrated including the 30S S21 protein of *L. sakei* against *Listeria monocytogenes*; the 50S L27 and L30 proteins of *L. salivarius* against *Streptococcus pyogenes*, *Streptococcus uberis*, and *Enterococcus faecium*; and the 50S L36 protein of *Pediococcus acidilactici* against *E. coli* and *Listeria innocua* [44,49,69]. On the other hand, it was described that *Bacillus tequilensis* secretes the 50S L1 protein with inhibitory action against *S. aureus* [45]. In the *Lactiplantibacillus* genus, only the proteins 50S L36, with activity against various phytosanitary pathogens, and 50S L14, with inhibitory activity against *Salmonella*, *E. coli*, *Listeria*, and *Weissella*, have been described [46,70]. Of note, the latter protein was detected in the CRL681 strain. Taken together, these findings reinforce the idea that both peptidoglycan hydrolases and ribosomal proteins could contribute in a complementary manner to the antagonistic effect of *L. plantarum* CRL681 against pathogenic bacteria. However, one limitation of our study is the lack of experimental validation of the functions of the specific proteins identified. Therefore, further studies are required to confirm their antimicrobial activity in the CRL681 strain against *E. coli* O157:H7.

In addition to the potential antimicrobial compounds identified, *L. plantarum* CRL681 presented several adhesion-associated proteins, most of which correspond to key enzymes in cellular metabolism that, through mechanisms not yet fully understood, are capable of localizing to the bacterial surface and performing adhesion functions. These include GAPDH, enolase, triose phosphate isomerase, phosphoglycerate kinase, glucose-6-phosphate isomerase, pyruvate kinase, lactate dehydrogenase, fructose bisphosphate aldolase, elongation factor Tu (EF-Tu), and stress response-related proteins such as GroEL and DnaK. These proteins have previously been identified on the cell surface of different lactobacilli species [25,35,36]. The expression of these adhesion factors could favor interaction with the intestinal mucosa or with the meat surface, contributing to the competitive exclusion of pathogens in both food and the gastrointestinal tract. Beyond their antagonistic activity, peptidoglycan hydrolases and ribosomal proteins also exhibit immunomodulatory functions [48,56], suggesting that they may contribute to the ability of *L. plantarum* CRL681 to modulate the innate immune responses in the intestinal mucosa [10,11]. The precise role of these specific proteins in the probiotic properties of the CRL681 strain should be experimentally validated in future research.

This study also investigated the differential expression of proteins in *L. plantarum* CRL681 during co-culture with EHEC. The study confirmed the metabolic adaptation of the CRL681 strain during microbial competition. In its exoproteome, a decrease in the expression of proteins involved in metabolite transport was observed, possibly to avoid the overexpression of unnecessary transport systems under limiting conditions. In addition, adjustments in central metabolism and energy production were evident, reflected in the reduction of proteins such as LdhL (lactate dehydrogenase), key enzymes in sugar catabolism (GapB and PgK), and thiamine biosynthesis (ApbE1). These findings suggest a metabolic regulation strategy to prioritize energy efficiency under competitive stress. Likewise, the decrease in the expression of peptidases such as PbpX2 and DacA, involved in peptidoglycan synthesis, could reflect an energy-saving mechanism in cell envelope biosynthesis or an adaptive response to bacterial competition. On the other hand, in the surfaceome, overexpression of proteins related to stress response was evident. In fact, glutathione reductase (GshR2) showed an almost eightfold increase, indicating an active response to oxidative stress generated by the presence of the pathogen. At the same time, acetate kinase (Ack2) increased 1.8-fold, highlighting a reconfiguration of carbon metabolism. This enzyme participates in the production of acetyl phosphate, a key energy intermediate in fermentation and mixed acid production, whose activity varies according to the availability of the carbon source in the environment. This data suggests that *L. plantarum* CRL681 adjusts its metabolism to optimize energy production in a competitive environment. The reduction of the ribose-5-phosphate isomerase (RpiA1) suggests an adjustment in the pentose phosphate pathway, possibly to redirect metabolic flux according to nutrient availability. The lower expression of ATP synthase gamma chain (AtpG) and Mn-dependent pyrophosphatase (PpaC) indicates an adaptation in energy production, probably in response to a more anaerobic microenvironment induced by *L. plantarum* CRL681 in the presence of *E. coli*. Overall, proteomic analysis of the co-culture of *L. plantarum* CRL681 and *E. coli* NCTC12900 reveals an adaptive advantage reflected in an efficient metabolic and physiological response by the lactic acid bacterium strain, allowing it not only to maintain its viability but also to inhibit EHEC.

## 4. Materials and Methods

### 4.1. Bacterial Strains and Culture Conditions

*Lactiplantibacillus plantarum* CRL681 belongs to the CERELA-CONICET (Tucuman, Argentina) culture collection and was isolated from Argentine fermented sausages [71]. The cultures were obtained from stocks stored at −80 °C by transferring three times in MRS broth (Merck, Buenos Aires, Argentina) and incubating at 30 °C for 24 h. The last subculture was incubated overnight (O/N). *Escherichia coli* O157:H7 NCTC12900, (National Type Culture Collection, Colindale, London, UK) is a natural mutant incapable of producing the enterotoxins Stx1 or Stx2 [72,73]. The culture was obtained by performing three transfers in Luria Bertani (LB) medium and incubating at 37 °C with shaking for 24 h. The last subculture was incubated O/N.

### 4.2. Chemically Defined Medium

A CDM was used to avoid protein interference during protein analysis. All chemicals used—amino acids, nitrogenous bases, vitamins, sugars, and inorganic salts—were obtained with the highest degree of purity available from Sigma-Aldrich (St. Louis, MO, USA). The CDM was prepared according to previously published works [74], from individual concentrated stock solutions, which were stored at 4 °C and adjusted to a final pH of 6.5. A final concentration of 0.5% glucose was used as the carbon source. Finally, the medium was sterilized in an autoclave and thereafter supplemented with the thermolabile components (vitamins) previously sterilized by filtration through a cellulose acetate membrane with a pore size of 0.22 µm (Sartorius AG, Goettingen, Germany). Once the medium was prepared, it was divided according to the volume required for the different tests.

### 4.3. Exoproteome Study: Isolation of Proteins Secreted into the Culture Medium

The exoproteome was obtained according to previous works [75], with some modifications. To analyze the exoproteome of *L. plantarum* CRL681 in the presence of the pathogen (co-culture), 200 mL of CDM was inoculated with 1 × 10^6^ CFU/mL of lactobacilli and 1 × 10^4^ CFU/mL of EHEC. After 24 h of incubation at 30 °C, the culture was centrifuged at 8000 rpm for 15 min at 4 °C, and the supernatant was filtered through Nalgene MF75 series polyester sulfone (PES) membrane filters with 0.22 µm pores to remove the remaining cells. Trichloroacetic acid (TCA) 12% (*w*/*v*) was added to the filtrate and left O/N at 4 °C. The protein precipitate was recovered by centrifugation (11,000 rpm for 30 min at 4 °C) and washed twice in cold acetone. Once the acetone had been removed by evaporation at room temperature, the precipitate containing the proteins was solubilized in 100 µL of sample buffer (7 M urea, 2 M thiourea, and 1% CHAPS). The total protein concentration was quantified using Bradford reagent. The samples were stored at −80 °C until use. For the controls, 100 mL of CDM was separately inoculated with *L. plantarum* CRL681 (10^6^ CFU/mL) and 100 mL of CDM with *E. coli* (10^4^ CFU/mL) for 24 h. To emulate the decrease in EHEC viability produced in the presence of lactic acid bacterium, the EHEC control culture was treated with lactic acid (10%), simulating the decrease in pH and controlling viability to obtain kinetics similar to those obtained in the co-culture (EHEC + CRL681). Once incubation was complete, both control cultures were combined and treated as a single sample, following the steps described above. Finally, the samples were subjected to SDS-PAGE, and the volume corresponding to 20 µg of proteins from each condition was mixed with 1/3 volume of 4× Laemmli buffer. Three independent assays were performed to obtain the triplicates required for proteomic analysis.

### 4.4. Surfaceome Study: Enzymatic Digestion of the Cell Surface

Surface proteins were digested according to the method previously described [76], with some modifications. The expression of surface proteins of *L. plantarum* CRL681 in the presence of *E. coli* was determined by inoculating the lactic acid bacterium (10^6^ CFU/mL) and EHEC (10^4^ CFU/mL) in 20 mL of CDM. After 24 h of incubation at 30 °C, the culture was centrifuged (8000 rpm for 15 min at 4 °C), the supernatant was discarded, and the pellet containing the cells was washed three times with PBS and resuspended in 500 µL of PBS with 30% sucrose (pH: 7.2). Enzymatic digestion was performed with 5 µg/mL trypsin to release the proteins from the cell wall and membrane. The samples were incubated for 30 min at 37 °C with agitation. After this time, the samples were centrifuged (12,000 rpm for 15 min at 4 °C) and the supernatant was filtered with a Sartorius Minisart RC15 syringe filter with a pore size of 0.22 µm to remove the remaining bacterial cells. The filtrates containing the surfaceome were frozen and lyophilized. For the controls, 20 mL of CDM were separately inoculated with *L. plantarum* CRL681 (10^6^ CFU/mL) and 20 mL of CDM with *E. coli* (10^4^ CFU/mL) for 24 h. Considering the antagonistic effect of the lactic acid bacterium on the pathogen, the viability of *E. coli* was controlled by adding lactic acid (10%), simulating the decrease in pH and cell count as if it were in co-culture with *L. plantarum* CRL681. Once incubation was complete, both cultures were combined and treated as a single sample, following the steps described above. Cell viability was checked before and after treatment with trypsin to detect possible cell lysis. The samples were analyzed by mass spectrometry at the CEQUIBIEM Proteomics Centre (QB-FCEN-UBA/IQUIBICEN-CONICET).

### 4.5. Samples Processing for Proteomic Analysis

The samples were reduced by 20 mM DTT for 45 min at 56 °C and alkylated with 50 mM iodoacetamide for 45 min in the dark. They were then digested with trypsin overnight to cleave the carboxyl end of lysine and arginine residues. Peptide extraction was performed with acetonitrile. The samples were lyophilized using Speed Vac (ThermoScientific, Waltham, MA, USA) and resuspended in 10 µL of 0.1% formic acid. ZipTip C18 tips (Merck) were used for desalting. The analysis was performed using nanoHPLC coupled to a ThermoScientific Orbitrap mass spectrometer (Waltham, MA, USA), model EASY-nLC 1000, designed to separate protein complexes with a high degree of resolution. A reverse-phase column was used for this purpose, which first allowed the separation of the peptides obtained by tryptic digestion and then their identification. The samples were ionized using electrospray. The data obtained was analyzed using Proteome Discoverer software (Thermo Scientific, version 2.2). The search criteria were as follows: Database: *L. plantarum* UP000000432, *E. coli* O157:H7 UP000000558, enzyme: trypsin, miscleavage: 2, mass tolerance for precursor: 10 ppm, mass tolerance for fragment: 0.05 Da, dynamic modifications oxidation (M), static modifications carbamoyl methylation (C), peptide confidence level: high, minimum number of different peptides identified per protein: 2.

To detect changes in the expression of *L. plantarum* CRL681 proteins between the monoculture and the co-culture with *E. coli* O157:H7, a label-free quantitative proteomic analysis was performed using three independent replicates per experimental condition. Significant differences were determined using Perseus software (Max Planck Institute of Biochemistry, Bavaria, Germany, version 1.6.6.0). A two-tailed Student’s *t*-test was applied to the quantification values of each protein. Changes were considered statistically significant if proteins simultaneously met the following criteria: *p*-value < 0.05, False Discovery Rate (FDR) ≤ 0.05, and a minimum fold-change of ±1.5 between conditions.

### 4.6. Bioinformatic Analysis

According to the information obtained from the exoproteome and surfaceome studies of *L. plantarum* CRL681, a possible conformation of the secretome for this strain was proposed. To this end, the identified proteins were manually filtered according to the following criteria: their presence in the in silico *L. plantarum* CRL681 secretome and their cellular localization, determined using the PSORT and DeepLocPro programs. Due to the variability observed in relation to localization, those proteins that showed extracellular, membrane, cell wall, or multilocation in one or both programs were included. Proteins with cytoplasmic or unknown localization in both programs were included if they met the following criteria: their secretion pathway was associated with a non-classical or signal peptide-mediated mechanism, or if they were proteins with moonlighting functions. In addition, protein functionality, available bibliographic evidence, and support in databases such as UniProt and InterPro were considered.

The presence of signal peptides in secreted proteins was determined using SignalP (SignalP 6.0—DTU Health Tech—Bioinformatic Services) [77]. The identified proteins were functionally annotated using COGclassifier v1.0.5 and EggNOG V5.0 to classify proteins into COG functional categories [78]. PSORT v3.0.3 (PSORTb Subcellular Localization Prediction Tool, version 3.0) [79] and DeepLocPro, version 1.0 (DeepLocPro 1.0, DTU Health Tech, Bioinformatic Services, Kongens Lyngby, Denmark) [80] were used to determine cellular localization. Secreted proteins were grouped into functional protein association networks using STRING v12.0. The network was constructed by representing the proteins identified in the subproteomes, removing those that showed no interactions, and highlighting those with differential expressions. In this network, each protein is represented as a node, while the connecting lines indicate functional associations. The thickness of the lines reflects the confidence level of the interaction according to the prediction sources integrated by STRING (databases, experimental data, gene fusion, co-occurrence, textmining, co-expression, and neighborhood). A high confidence level (0.700) was applied (STRING: functional protein association networks) [81]. MoonProt v3.0 (MoonProt, A Database for Moonlighting Proteins) [82] was used to identify proteins with moonlighting functions in *L. plantarum* CRL681 secretome. For comparative genomics analyses, the Venn diagram was created using the SRplot web server [83]. The similarity of the extracellular protein sequence between *L. plantarum* CRL681 and *L. plantarum* WCFS1 (type strain) genomes was determined by BLASP versions 2.16.0.

## 5. Conclusions

This study is the first to define the exoproteome and surfaceome of *L. plantarum* CRL681 using an integrated functional genomics and proteomics approach with state-of-the-art technologies. The proposed secretome involves proteins that may be directly or indirectly associated with the demonstrated antagonistic capacity of the CRL681 strain against EHEC, as well as with its probiotic properties. Proteomic analyses revealed the presence of peptidoglycan hydrolases capable of degrading the cell wall of EHEC, suggesting a direct mechanism of action affecting pathogen viability. In addition, extracellular ribosomal proteins known for their antimicrobial properties were identified, whose interaction with bacterial membranes could lead to lipid redistribution, pore formation, and subsequent leakage of intracellular components. Beyond their antagonistic activity, peptidoglycan hydrolases and ribosomal proteins also exhibit immunomodulatory functions, suggesting that they may contribute to the ability of *L. plantarum* CRL681 to stimulate the mucosal innate immune responses.

The differential expression of proteins during the coculture of *L. plantarum* CRL681 with EHEC revealed strategic adaptations of the lactic acid bacterium that enable its competitive advantage and strong inhibitory activity. Therefore, the presence and abundance of these proteins in the *L. plantarum* CRL681 secretome suggest that its competitive success and antagonistic effect against EHEC are mediated by synergistic mechanisms involving nutrient competition, stress resistance, antimicrobial compound production, and surface adhesion. These findings support the potential of *L. plantarum* CRL681 as a bioprotective agent in foods and as a possible functional probiotic.

## Figures and Tables

**Table 1:** Proteins with moonlighting function identified in the exoproteome of *L. plantarum* CRL681.

Access	Name	Moonlighting Function	Reference
F9UM10	Glyceraldehyde 3-phosphate dehydrogenase	Adhesion to plasminogen, actin, myosin, and fibronectin	[30]
Q88VE0	Elongation factor Tu	Adhesion to plasminogen, fibrinogen, laminin, and actin	[31]
Q88YH5	Phosphoglycerate kinase	Plasminogen and actin adhesion	[32]
Q88YH3	Enolase	Fibronectin adhesion	[33]
F9US66	Serine protease Htra	Fibronectin adhesion	[34]
Q88YH4	Triosaphosphate isomerase	Adherence to Caco-2 cells, competition by exclusion	[35]
F9UTT2	Fructose bisphosphate aldolase	Adhesion	[36]
Q88VJ2	Lactate dehydrogenase	Adhesion to Caco-2 cells,	[37]
F9UMI2	Cold Shock Protein (CspC)	Adhesion	[38]

**Table 2:** Moonlighting proteins identified in the surfaceome of *L. plantarum* CRL681.

Access	Name	Moonlighting Function	Bibliography
Q88VE0	Elongation factor Tu	Actin and plasminogen adhesion	[31]
F9UPM3	Pyruvate kinase	Actin and plasminogen adhesion	[32]
F9UTT2	Fructose bisphosphate aldolase	Actin and plasminogen adhesion	[36]
Q88UI4	Glucose 6 phosphate isomerase	Adhesion to laminin and collagen	[39]
Q88XY8	Elongation Factor G	Mucin adhesion	[36]
Q88YH4	Triosaphosphate isomerase	Caco-2 cell adhesion	[35]
F9UNS5	6-phosphogluconate dehydrogenase	Laminin adhesion	[40]
Q88YH3	Enolase 1	Fibronectin adhesion	[33]
F9UTA3	Inosine 5′ monophosphate (IMP)	Fibronectin adhesion	[37]
Q88YM5	Chaperone GroEL	Mucin adhesion	[41]
Q88YH5	Phosphoglycerate kinase	Plasminogen adhesion	[32]
Q88VM0	Chaperone DnaK	Plasminogen adhesion	[42]
Q88VJ2	D-lactate dehydrogenase	Fibronectin adhesion	[37]
F9UM10	Glyceraldeheid 3-phosphate dehydrogenase	Plasminogen adhesion	[30]
F9UQ92	Pyruvate dehydrogenase	Fibronectin adhesion	[43]
Q88VY1	ATP-dependent 6-phosphofructokinase	Adherence to invertase	[37]
Q88WN3	50S ribosomal protein L27	Antimicrobial	[44]
Q88XW8	50S ribosomal protein L30	Antimicrobial	[44]
Q88YW9	50S ribosomal protein L1	Antimicrobial	[45]
Q88VD5	30S ribosomal protein S15	Antimicrobial	[32]
Q890J8	50S ribosomal protein L9	Antimicrobial	[32]
Q88XX6	50S ribosomal protein L14	Antimicrobial	[46]
Q88WN5	50S ribosomal protein L21	Antimicrobial	[47]

## Data Availability

Mass spectrometry proteomic data have been deposited in the ProteomeXchange Consortium via the associated repository PRIDE [84,85] with the dataset identifier PXD059813.

## Supplementary Materials

The following supporting information can be downloaded at https://www.mdpi.com/article/10.3390/antibiotics15010096/s1. Supplementary Figure S1. Interaction network of proteins identified in the surfaceome of *Lactiplantibacillus plantarum* CRL681 during the co-culture with *Escherichia coli* O157:H7 NCTC12900. The colors in the node halos indicate a decrease (red) or increase (blue) in protein expression. The intensity of the color indicates the level of expression. In addition, the different colors in the nodes represent distinct functionality: blue nodes (ribosomal proteins), gray nodes (redox homeostasis), yellow nodes (carbon metabolism), magenta and lilac nodes (ABC-type transporters), light blue nodes (oxidative phosphorylation), light green and orange nodes (chaperones and stress response proteins), purple nodes (cell division and cell wall formation), and brown nodes (proteins identified as stress response through bibliographic sources) (high resolution format of Figure 5). Supplementary Table S1: In silico secretome of *L. plantarum* CRL681, Supplementary Table S2: In vitro exoproteome of *L. plantarum* CRL681. Supplementary Table S3: List of proteins identified with decreased expression in the exoproteome of *L. plantarum* CRL681 during coculture with EHEC in CDM at 24 h, at 30 °C, Supplementary Table S4: In vitro surfaceome of *L. plantarum* CRL681, Supplementary Table S5: *L. plantarum* CRL681 proteins identified with differential expression in coculture with *E. coli* NCTC12900 in CDM at 30 °C, Supplementary Table S6: Proposed in vitro secretome for *L. plantarum* CRL681, Supplementary Table S7: Secretome proteins involved in the competitive advantage of *L. plantarum* CRL681 against EHEC. The proteins highlighted in pink indicate that they were identified in both proteomes (surfaceome and exoproteome), Supplementary Table S8: Viability of *L. plantarum* CRL681 before and after cell shaving performed with 5 µg/mL trypsin at 37 °C for 30 min under agitation.
